# Diagnostic MicroRNA Biomarker Discovery for Non-Small-Cell Lung Cancer Adenocarcinoma by Integrative Bioinformatics Analysis

**DOI:** 10.1155/2017/2563085

**Published:** 2017-06-15

**Authors:** Yang Shao, Bin Liang, Fei Long, Shu-Juan Jiang

**Affiliations:** Department of Respiratory Medicine, Shandong Provincial Hospital Affiliated to Shandong University, Jinan, Shandong 250021, China

## Abstract

Lung cancer is the leading cause of cancer death and its incidence is ranked high in men and women worldwide. Non-small-cell lung cancer (NSCLC) adenocarcinoma is one of the most frequent histological subtypes of lung cancer. The aberration profile and the molecular mechanism driving its progression are the key for precision therapy of lung cancer, while the screening of biomarkers is essential to the precision early diagnosis and treatment of the cancer. In this work, we applied a bioinformatics method to analyze the dysregulated interaction network of microRNA-mRNA in NSCLC, based on both the gene expression data and the microRNA-gene regulation network. Considering the properties of the substructure and their biological functions, we identified the putative diagnostic biomarker microRNAs, some of which have been reported on the PubMed citations while the rest, that is, miR-204-5p, miR-567, miR-454-3p, miR-338-3p, and miR-139-5p, were predicted as the putative novel microRNA biomarker for the diagnosis of NSCLC adenocarcinoma. They were further validated by functional enrichment analysis of their target genes. These findings deserve further experimental validations for future clinical application.

## 1. Introduction

Lung cancer is the most death causing cancer for both men and women in the United States, and it is also the most death causing cancer in men and second in women worldwide. The incidence rate is high and ranked second for both men and women in the United States [1, 2]. Non-small-cell lung cancer (NSCLC) adenocarcinoma is one of the most common histological subtypes of lung cancer [3] and it is reported that nearly 40% of the lung cancer are adenocarcinoma, and the other two subtypes of NSCLS are squamous-cell carcinoma and large-cell carcinoma [4]. The NSCLS adenocarcinoma is reported associated with the aberrations like the epidermal growth factor receptor (EGFR) mutations and anaplastic lymphoma kinase (ALK) fusion or rearrangement [3, 5], and several drugs such as gefitinib, erlotinib, and afatinib were developed for the targeting the aberrant gene products, but only few patients are ideal for the targeted treatments [6]. In addition, the patients treated with these target drugs may acquire resistance and make the treatment invalid [7]. There are also other aberrations reported associated with the NSCLC adenocarcinoma, such as mutations or fusions happen in HER2, BRAF, NF1, MEK1, RET, ROS1, and other genes. The risk factors for NSCLS adenocarcinoma may also include air pollution, gender, age, smoking, occupation, and eating habits [8–10]. For personalized diagnosis and treatment of cancer, the expression profile characterization and the key player screening [11] are the necessary steps. With the coming of aging era and the air pollution in the developing countries, the incidence of lung cancer will keep high, and the early diagnosis of lung cancer becomes very necessary. However, we still lack sensitive and precision biomarkers for the early diagnosis or the personalized therapy of the lung cancer [7, 12, 13].

MicroRNAs are endogenous small noncoding RNAs which regulate many important biological roles and theirs aberrations may have significant effects on the cancer genesis and progression, such as cell proliferation, cell cycle, apoptosis, and tumorigenesis, and therefore are good candidates for cancer diagnosis and therapy biomarkers [14–16]. Biomarker microRNA discovery could be implemented both experimentally and computationally. The former is a routine but time-consuming and costing method, since the biological systems are complex and the mechanisms are diverse. The computational methods based on integrative analysis of different omics data have more advantages, such that it could integrate diverse omics data sets and model the biological process by network construction and then understand the aberrations at the systems level [17, 18]. Furthermore, the computational methods are also cheap, less time-consuming and could be easily validated by literature mining, association analysis, and bioinformatics functional enrichment confirmation. With more and more biomedical data available and accumulated, the computational methods will be more and more powerful for the future precision medicine strategies.

At present, more and more computational and bioinformatics models are developing for biomarker discovery; some of them are machine learning based [19, 20], while others are mechanism-based [14–16, 21]. The machine learning based methods need more data to train the model and the mechanism-based models are more knowledge based, and the two types of methods complement each other and promote biomarker discovery. We here applied the previous reported mechanism-based method, which is successful in microRNA biomarker discovery, to screen novel diagnostic biomarker for NSCLC adenocarcinoma.

## 2. Materials and Methods

**Figure 1:**
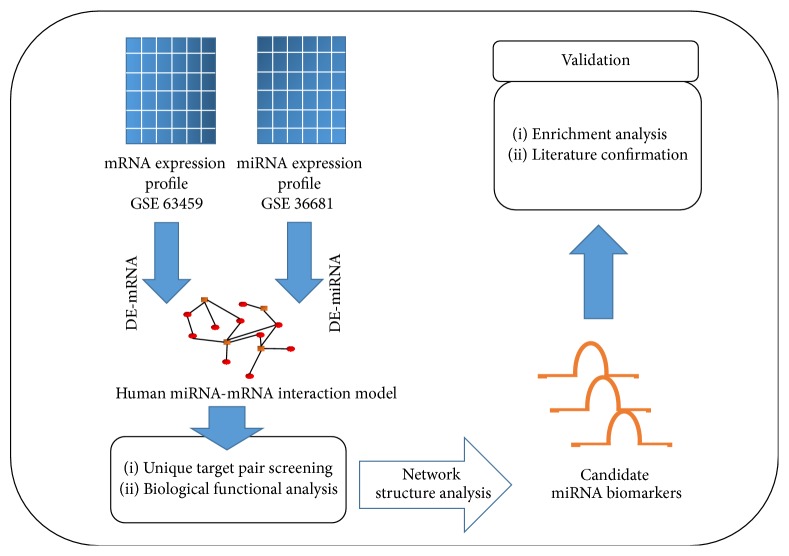
The procedure of data collection, identification of microRNA biomarkers with integrative analysis, and the validation of the miRNA biomarkers.

The data used in our integrative analysis include the gene expression data of NSCLC adenocarcinoma from both microRNA and messenger RNA(mRNA) and the human reference microRNA-mRNA network. We need to first collect the data and reconstruct the NSCLC adenocarcinoma specific network and then to identify putative microRNA biomarkers based on the network structure and their biological functions. The screened microRNAs need to be validated by literature mining, confirmation, and bioinformatics exploration of their associations with NSCLC adenocarcinoma. The pipeline of the whole process of this work could be seen in Figure 1.

### 2.1. The NSCLC Adenocarcinoma Gene Expression Data Collection and the Human Reference MicroRNA-mRNA Interactions

The gene expression and microRNA expression data for the NSCLC adenocarcinoma were extracted from the public GEO database [22]. The details of data sets are listed in Table 1. The data sets we used for the construction of NSCLC adenocarcinoma specific microRNA-mRNA network are GSE63459 and GSE36681, where the GSE63459 data set is the mRNA expression data which includes 33 NSCLC adenocarcinoma samples and 32 samples as control, and the other data set is the microRNA expression data with 47 NSCLC adenocarcinoma samples and 47 control samples. The data were first normalized and the differentially expressed mRNAs were then selected with the linear models in limma R package [23, 24]. The empirical Bayes (eBayes) method was applied to calculate the *p* value and other parameters. The Benjamini-Hochberg correction was used to adjust the *p* values. The adjusted *p* values less than 0.05 were regarded as significant. The human reference microRNA-mRNA interaction network was constructed based mainly on experimentally validated and the consensus predicted microRNA-mRNA interaction pairs as reported in previous studies [16, 25, 26].

### 2.2. Model Construction, Validation, and Identification of NSCLC Adenocarcinoma MicroRNA Biomarkers

The basic idea and the methods we used here are based on the models which were developed previously [26, 40–42], where two parameters are used to measure the importance of microRNAs as the potential biomarkers for a specific disease. The first is the number of genes (NOG) uniquely targeted by a certain microRNA [40, 41], and this index is reasonable to quantify the tendency to be a biomarker since the alteration of the unique interaction cannot be substituted or compensated by other microRNA-mRNA interaction pairs. The other index was proposed to quantify the transcription factor percentage (TFP) and was defined as the percentage of transcription factor (TF) genes of all the microRNA targets [26]. With these two indexes, the NSCLC adenocarcinoma specific microRNA-mRNA interaction network was constructed by mapping the detected differentially expressed microRNAs in NSCLC adenocarcinoma onto the reference human microRNA-mRNA interaction network. With the reconstructed conditional network, the abovementioned measurements, that is, the NOG and TFP, were calculated for each microRNA in the NSCLC adenocarcinoma network. MicroRNAs with significant large NOG and TFP values (Wilcoxon signed-rank test, *p* value < 0.05) were detected as our putative biomarkers for diagnosis of NSCLC adenocarcinoma.

To validate the bioinformatics model first, we also collected reported NSCLC adenocarcinoma associated microRNAs from PubMed citations with the searching criteria “(lung adenocarcinoma OR NSCLC adenocarcinoma) AND (miRNA OR microRNA) AND (biomarker^*∗*^  OR marker^*∗*^)”. The related PMIDs, NOGs, and TFPs of these microRNAs were also calculated.

### 2.3. The Literature Confirmation and Functional Validation of the Putative NSCLC Adenocarcinoma Diagnostic MicroRNA Biomarkers

For validation of the bioinformatics method and the screened microRNA biomarkers, we checked the PubMed citations and extracted the reported microRNA biomarkers for both diagnosis and prognosis of NSCLC adenocarcinoma. The identified novel microRNA biomarkers were then validated with functional enrichment analysis of the targeted genes of the microRNAs. The enrichment analysis were performed with Gene Ontology Annotations, KEGG pathway analysis, which were done by the DAVID (Database for Annotation, Visualization, and Integrated Discovery) online tool [43]. The *p* value threshold was set to 0.05 and the FDR adjustment was used for multiple test correction. Then we calculated the enrichment based on the hypergeometric test.

## 3. Results and Discussion

### 3.1. The Validation of Bioinformatics Methods for the MicroRNA Biomarker Discovery in NSCLC Adenocarcinoma

The microRNA biomarkers for diagnosis and prognosis of NSCLC adenocarcinoma reported in PubMed citations were collected and listed in Table 2. According to the NOGs and TFPs listed in Table 2, all the microRNAs except miR-650 are characterized with high NOGs and TFPs, and therefore the performance of the microRNA biomarker discovery method based on the two measurements is reasonable and can be extended and applied to the biomarker discovery in NSCLC adenocarcinoma.

Among the reported microRNA biomarkers for NSCLC adenocarcinoma, many of them played essential roles in lung carcinogenesis and their abnormal expression patterns were highly associated with the occurrence and development of NSCLC adenocarcinoma. For example, serum miR-155 was a sensitive indicator for predicting the initiation of lung adenocarcinoma, especially combining with the index of carbohydrate antigen 125. It altered the expression of downstream proteins and activated the lung carcinogenic signal [27]. Two miRNAs, that is, miR-196a-5p and miR-218-5p, were validated to be up- and downregulated from normal to adenocarcinoma tumor tissues, respectively. Their target genes were functional in lung cancer related processes by activating or inhibiting biological activities in pathways in cancer, cell cycle, transcriptional misregulation in cancer, and small-cell lung cancer [28]. The prognostic value of miRNAs for NSCLC adenocarcinoma was also comprehensively investigated. For instance, Huang et al. [31] showed that miR-650 was able to regulate the expression of Bcl-2/Bax, which would thereby contribute to the docetaxel chemoresistance of lung adenocarcinoma cells. This miRNA was a powerful indicator for predicting the chemosensitivity of lung adenocarcinoma patients to docetaxel-based chemotherapy regimen. Zhang et al. [32] analyzed the clinical potential of miR-141 and found that this miRNAs was positively correlated with the tumor size, lymph NOGe metastasis, and TNM stage of lung adenocarcinomas. Meanwhile, Liu et al. [34] screened that the upregulation of plasma exosomal miRNAs miR-23b-3p, miR-10b-5p and miR-21-5p was strongly connected with the poor overall survival of lung adenocarcinoma patients. In order to evaluate the efficacy of maintenance treatment on lung adenocarcinoma patients with negativity for epidermal growth factor receptor (EGFR) mutations or anaplastic lymphoma kinase (ALK) translocations, Shi et al. [36] designed the experiment in which patients were divided into a pemetrexed group and a control group, respectively. As a result, the expression levels of miR-25, miR-145, and miR-210 were associated with the progression-free survival time of patients in the treatment group, which highlighted the prognostic potential of these miRNAs to the pemetrexed therapy in specific lung adenocarcinoma individuals.

### 3.2. The Predicted MicroRNA Biomarker for Diagnosis of NSCLC Adenocarcinoma

We performed the predictions according to the pipeline shown in Figure 1. At first, 93 differentially expressed microRNAs and 331 differentially expressed genes in NSCLC adenocarcinoma were detected, respectively. Nine microRNAs were screened by Wilcoxon signed-rank test with *p* value < 0.05. These nine microRNAs were predicted to be biomarkers for the diagnosis of NSCLC adenocarcinoma as listed in Table 3. Their network structural features in the microRNA-mRNA interaction network were shown, which are the whole set of targeted genes, NOGs and TFPs. Four of the nine predicted microRNAs (bolded and underlined in Table 3), that is, miR-145-5p, miR-182-5p, miR-141-3p, and miR-590-3p, have been reported as biomarkers previously, the remaining five, that is, miR-204-5p, miR-567, miR-454-3p, miR-338-3p, and miR-139-5p, were recommended as novel diagnosis biomarker for NSCLC adenocarcinoma.

### 3.3. Functional Enrichment Validation of the Predicted MicroRNA Biomarkers

We further performed the functional enrichment analysis to investigate the roles of genes regulated by identified microRNA biomarkers through Database for Annotation, Visualization and Integrated Discovery (DAVID) online tools. This analysis was conducted in two ways: Gene Ontology (GO) analysis and KEGG pathway analysis.

**Figure 2:**
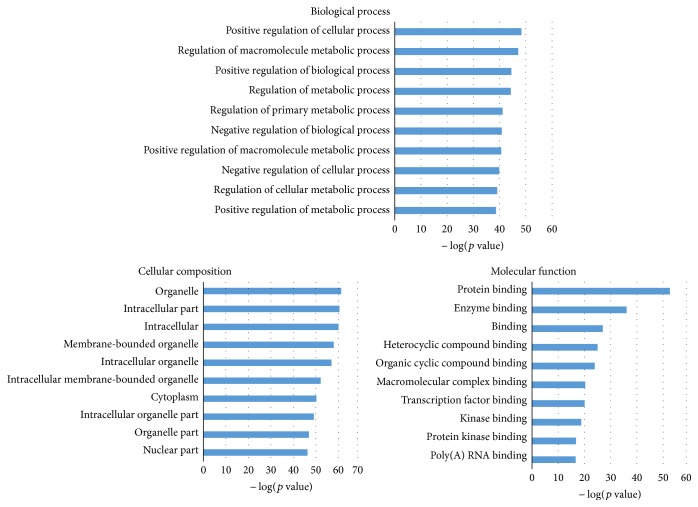
Gene ontology (GO) analysis for genes targeted by 9 identified microRNA biomarkers. The statistical significance value (*p* value) has been negative 10-based log transformed. Top 10 significantly enriched items are listed for each level.

In GO analysis, we did this analysis at three levels: biological process (BP), cellular component (CC), and molecular function (MF). The top 10 most significantly enriched items were shown in Figure 2. Through further literature validation, we found that most of the items have a strong relationship with lung adenocarcinoma. For example, in molecular function level, relevant research has shown that FGFR2's function could be regulated by two proteins: Grb2 and Plc*γ*1 under the situation of growth factor absence. These two proteins will compete for the same protein binding site [44]. FGFR2 expression could be repressed by miR-338-3p, one of the identified miRNA biomarkers. Other items, such as cytoplasm [45], membrane-bounded organelle [46], positive regulation of metabolic process, and transcription factor binding [47, 48] have already been validated through biological and clinical experiments to have an impact on the occurrence and metastasis of lung adenocarcinoma.

**Figure 3:**
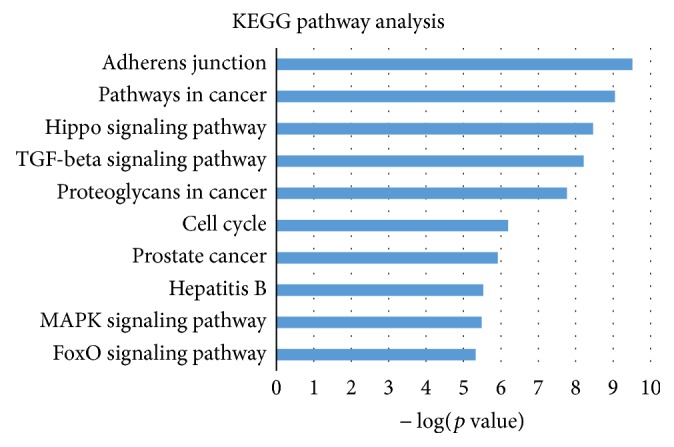
KEGG pathway enrichment analysis for genes targeted by 9 candidate microRNA biomarkers. The statistical significance value (*p* value) has been negative 10-based log transformed. The top 10 significantly enriched pathways are listed, respectively, in this figure.

In KEGG pathway analysis, we totally found 72 significantly enriched pathways. Here we still selected top 10 most significantly enriched pathways for further investigation. These 10 pathways were listed in Table 4 and shown in Figure 3.

**Figure 4:**
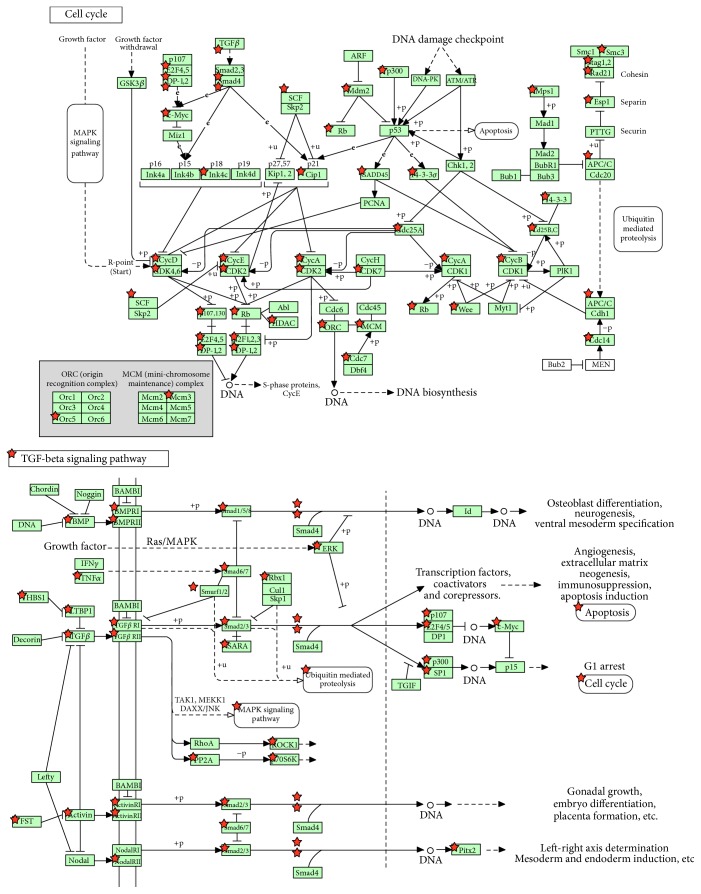
The pipeline of cell cycle and MAPK signaling pathway. The stars in these pipelines represent the action sites of the genes regulated by 9 candidate miRNA biomarkers.

Here, we investigate the relationship between these 10 pathways and lung adenocarcinoma through literature validation and we found that most of these pathways have been demonstrated to be associated with lung adenocarcinoma. Besides common pathways in lung cancer like cell cycle and MAPK signaling pathways [49, 50], there are still other pathways such as Hippo signaling pathway [51] and TGF-beta signaling pathway and proteoglycans in cancer are all supported by relevant research [52, 53]. The relevant pipeline of cell cycle and MAPK signaling pathways can be referred to in Figure 4.

## 4. Conclusions

In this research, an integrative bioinformatics model considering the network structure and biological functions of the microRNA targets was used to predict novel biomarker microRNAs for the diagnosis of NSCLC adenocarcinoma. The method was first tested with the reported microRNA biomarkers of NSCLC adenocarcinoma; then we extended the model and applied it to the microRNA and gene expression data. We detected five novel biomarker microRNAs for the diagnosis of NSCLC adenocarcinoma, including miR-204-5p, miR-567, miR-454-3p, miR-338-3p, and miR-139-5p. The novel bioinformatics microRNAs were validated with bioinformatics exploring their functions associated with NSCLC adenocarcinoma.

## Figures and Tables

**Table 1:** NSCLC adenocarcinoma gene expression data collected from GEO data sets.

Accession/ID	PMID	Platform	Treatment	Control	Materials	Year	mRNA/miRNA
GSE36681	22573352	GPL8179	*n* = 47	*n* = 47	tissue	2012	miRNA
GSE63459	26134223	GPL6883	*n* = 33	*n* = 32	tissue	2015	mRNA

**Table 2:** Literature reported lung adenocarcinoma miRNA biomarkers.

Reported miRNA	Official ID	PMID	Biomarker type	Samples	Expression level	NOG	TFP
miR-155	miR-155-5p	24190459 [27]	Diagnosis	Serum	Up	71	0.21
miR-196a-5p	miR-196a-5p	27247934 [28]	Diagnosis	Tissue	Up	7	0.19
miR-218-5p	miR-218-5p	27247934 [28]	Diagnosis	Tissue	Down	10	0.12
miR-143	miR-143-3p	24286416 [29]	Diagnosis	Blood	Down	15	0.03
miR-182	miR-182-5p	19493678 [30]	Diagnosis	Tissue	Up	9	0.19
miR-650	miR-650	23991130 [31]	Prognosis	Tissue	Up	0	0
miR-141	miR-141-3p	25746592 [32]	Prognosis	Tissue	Up	22	0.17
miR-29c	miR-29c-3p	28241836 [33]	Prognosis	Tissue	Down	5	0.15
miR-23b-3p	miR-23b-3p	28055956 [34]	Prognosis	Plasma	Up	23	0.15
miR-10b-5p	miR-10b-5p	28055956 [34]	Prognosis	Plasma	Up	9	0.15
miR-21-5p	miR-21-5p	28055956 [34]	Prognosis	Plasma	Up	38	0.13
miR-126-3p	miR-126-3p	27277197 [35]	Prognosis	Tissue	Down	6	0.12
miR-451a	miR-451a	27277197 [35]	Prognosis	Tissue	Down	4	0.17
miR-25	miR-25-3p	26687391 [36]	Prognosis	Blood	Up	9	0.17
miR-145	miR-145-5p	26687391 [36]	Prognosis	Blood	Down	36	0.11
miR-210	miR-210	26687391 [36]	Prognosis	Blood	Down	1	0.18
miR-142-3p	miR-142-3p	23410826 [37]	Prognosis	Serum	Up	12	0.14
miR-29b	miR-29b-3p	22249264 [38]	Prognosis	Tissue	Down	9	0.14
miR-590	miR-590-5p	28012926 [39]	Prognosis	Tissue	Up	14	0.16

**Table 3:** Predicted putative lung adenocarcinoma microRNA biomarkers.

miRNA ID	NOG	*p* value	TFP	*p* value	Whole target genes
**miR-145-5p**	1	1.66*E* − 02	0.67	2.98*E* − 08	MMP12; ZFP36; KLF4

miR-204-5p	5	1.78*E* − 15	0.13	1.05*E* − 02	DPYSL2; EMP1; SPDEF; LMO7; SLC1A1; ALPL; MMP9; FRAS1

**miR-182-5p**	2	1.80*E* − 08	0.20	4.34*E* − 04	CAMK2N1; ZFP36; UBE2T; LPHN2; RGS17.

miR-567	1	1.66*E* − 02	0.25	4.61*E* − 04	SPTBN1; DUSP1; BCHE; LPHN2

**miR-141-3p**	2	1.80*E* − 08	0.14	2.05*E* − 03	H3F3B; TCEAL2; MYH10; LHFP; LPHN2; CCL2; KLF9

miR-454-3p	2	1.80*E* − 08	0.11	4.64*E* − 02	DPYSL2; HOXA5; FKBP11; SRPX; EDN1; LDLR; CAV2; BMPR2; SLC2A1

**miR-590-3p**	5	1.78*E* − 15	0.13	2.33*E* − 03	SERPINE2; PLEKHC1; SPTBN1; H3F3B; TMEM47; TIMP3; COL3A1; CXCL13; ETS2; CELSR3; LPL; SMAD6; BMPR2; LPHN2; SASH1.

miR-338-3p	2	1.80*E* − 08	0.25	4.61*E* − 04	COL1A1; FOSB; ADAMTS1; MMP9

miR-139-5p	1	1.66*E* − 02	1.00	1.49*E* − 08	FOS

**Table 4:** Top 10 significantly enriched pathways in KEGG pathway analysis.

Term	Adj. *p* value
Adherens junction	3.05*E* − 10
Pathways in cancer	8.91*E* − 10
Hippo signaling pathway	3.43*E* − 09
TGF-beta signaling pathway	6.13*E* − 09
Proteoglycans in cancer	1.72*E* − 08
Cell cycle	6.44*E* − 07
Prostate cancer	1.22*E* − 06
Hepatitis B	2.98*E* − 06
MAPK signaling pathway	3.29*E* − 06
FoxO signaling pathway	4.74*E* − 06

## Abbreviations

NSCLC: Non-small-cell lung cancer
DE: Differentially expressed
TF: Transcription factor
NOG: Novel out degree
TFP: Transcription factor gene percentage
KEGG: Kyoto encyclopedia of genes and genomes
DAVID: Database for Annotation, Visualization, and Integrated Discovery.
